# Activation of the *β*‐common receptor by erythropoietin impairs acetylcholine‐mediated vasodilation in mouse mesenteric arterioles

**DOI:** 10.14814/phy2.13751

**Published:** 2018-06-25

**Authors:** Cody R. Kilar, YanPeng Diao, Larysa Sautina, Sivakumar Sekharan, Shahar Keinan, Bianca Carpino, Kirk P. Conrad, Rajesh Mohandas, Mark S. Segal

**Affiliations:** ^1^ Division of Nephrology Hypertension, and Transplantation College of Medicine University of Florida Gainesville Florida; ^2^ Department of Physiology and Functional Genomics College of Medicine University of Florida Gainesville Florida; ^3^ Cloud Pharmaceuticals Inc. 6 Davis Dr Research Triangle Park North Carolina; ^4^ Department of Obstetrics and Gynecology College of Medicine University of Florida Gainesville Florida; ^5^ North Florida/South Georgia Veterans Health System Gainesville Florida; ^6^ Present address: The Cambridge Crystallographic Data Centre 174 Frelinghuysen Road Piscataway New Jersey 08854

**Keywords:** CD131, erythropoietin, hypertension, *β*‐common receptor

## Abstract

Clinically, erythropoietin (EPO) is known to increase systemic vascular resistance and arterial blood pressure. However, EPO stimulates the production of the potent vasodilator, nitric oxide (NO), in culture endothelial cells. The mechanism by which EPO causes vasoconstriction despite stimulating NO production may be dependent on its ability to activate two receptor complexes, the homodimeric EPO (EPOR_2_) and the heterodimeric EPOR/*β*‐common receptor (*β* CR). The purpose of this study was to investigate the contribution of each receptor to the vasoactive properties of EPO. First‐order, mesenteric arteries were isolated from 16‐week‐old male C57BL/6 mice, and arterial function was studied in pressure arteriographs. To determine the contribution of each receptor complex, EPO‐stimulating peptide (ESP), which binds and activates the heterodimeric EPOR/*β* CR complex, and EPO, which activates both receptors, were added to the arteriograph chamber 20 min prior to evaluation of endothelium‐dependent (acetylcholine, bradykinin, A23187) and endothelium‐independent (sodium nitroprusside) vasodilator responses. Only ACh‐induced vasodilation was impaired in arteries pretreated with EPO or ESP. EPO and ESP pretreatment abolished ACh‐induced vasodilation by 100% and 60%, respectively. EPO and ESP did not affect endothelium‐independent vasodilation by SNP. Additionally, a novel *β* CR inhibitory peptide (*β* IP), which was computationally developed, prevented the impairment of acetylcholine‐induced vasodilation by EPO and ESP, further implicating the EPOR/*β* CR complex. Last, pretreatment with either EPO or ESP did not affect vasoconstriction by phenylephrine and KCl. Taken together, these findings suggest that acute activation of the heterodimeric EPOR/*β* CR in endothelial cells leads to a selective impairment of ACh‐mediated vasodilator response in mouse mesenteric resistance arteries.

## Introduction

Since the introduction of recombinant erythropoietin (EPO) in the 1980s for treatment of anemia, it has become one of the most widely used cytokines in clinical practice (Jelkmann 1986; Tögel et al. 2016). A common adverse effect of EPO administration is elevation of arterial blood pressure and increase in systemic vascular resistance (Drüeke et al. 2006; Singh et al. 2006; Liu et al. 2011). While several factors, particularly an increase in erythrocyte mass, were initially postulated to cause EPO‐induced hypertension (Martin and Moncada 1988; Fishbane and Besarab 2007), a series of experiments by Vaziri et al. (1995) demonstrated that the hypertensive effects of EPO are independent of its erythropoietic action. While the erythropoietic action of EPO is mediated by the homodimeric EPO receptor (EPOR_2_), EPO also activates a heterodimeric EPOR/*β*‐common receptor (*β*CR), which is believed to be responsible for the tissue protective effects of EPO (Leist et al. 2004; Sautina et al. 2010; Coldewey et al. 2013; Yang et al. 2013). On one hand, EPO stimulates production of the potent vasodilator, nitric oxide (NO), in cultured bone marrow‐derived angiogenic cells (BMDACs) (Sautina et al. 2010) and endothelial cells (Su et al. 2011), via activation of the heterodimeric EPOR/*β*CR (Sautina et al. 2010). On the other hand, EPO also induces the release of the potent vasoconstrictor endothelin‐1 (ET‐1) in endothelial cells in vitro (Carlini et al. 1993a,b). EPO also directly acts on vascular smooth muscle cells to mobilize calcium and facilitate contraction (Morakkabati et al. 1996). To date, however, it has not been determined if the release of endothelin‐1 and effects on smooth muscle cell are mediated by the EPOR_2_ or the EPOR/*β*CR. Interestingly, in healthy human subjects, EPO administered intravenously into the brachial artery over a large concentration range impaired cyclooxygenase‐dependent vasodilation by acetylcholine in the forearm resistance vasculature without changing in blood pressure (Wada et al. 1999). To our knowledge, there has not been a study to date which identifies whether activation of the homodimeric EPOR_2_, the heterodimeric EPOR/*β*CR, or both is responsible for the vasoactive effects observed. The aim of this study was to evaluate whether the acute vasoactive effects of EPO are mediated by the homodimeric EPOR_2_, the heterodimeric EPOR/*β*CR, or both. To this end, we developed a novel *β*CR inhibitory peptide (*β*IP) (Kilar et al. 2018), which was extracted from a portion of helix‐A from a mutant EPO (PDB code: 1EER, chain A). We introduced a double mutation at sites 7 and 11 to the 16‐amino acid from the extracted portion of EPO and found the amino acid peptide model comprising of VLERYLHEAKHAEKIT decreases bioavailable nitric oxide and angiogenic potential of the *β*CR (Kilar et al. 2018). The use of this peptide allows us to study, in isolation, the effects of EPO on the heterodimer EPOR/*β*CR.

## Methods

The utilization of mice in this study was approved by the Institutional Animal Care and Use Committee at University of Florida and was in accordance with the National Institutes of Health *Guide for the Care and Use of Laboratory Animals* (revised 1996). Mice were housed in a 12:12‐h light/dark cycle and given access to standard mouse chow and water ad libitum.

### Drugs

Acetylcholine (ACh), bradykinin (BK), A23187, sodium nitroprusside (SNP), Tempol, phenylephrine (PE), and potassium chloride (KCl) were obtained from Sigma‐Aldrich (St. Louis, MO). Human recombinant erythropoietin (EPO) was purchased from Zydus (Cadila, India). EPO‐stimulating peptide (ESP) derived from a 11‐amino acid peptide sequence of the helix B of EPO which interacts with the *β*CR (Brines et al. 2008) and the *β*CR inhibitory peptide (*β*IP) were synthesized by the core facility (Kilar et al. 2018).

### Isobaric arteriography

Sixteen‐week‐old male C57BL/6 mice were anesthetized with 5% isoflurane/O_2_ mix and euthanized by exsanguination. The abdominal cavity was opened to expose the mesenteric cascade, which was then isolated and placed in ice‐cold HEPES‐buffered physiological saline solution (a modified Krebs buffer composed of sodium chloride 142 mmol/L, potassium chloride 4.7 mmol/L, magnesium sulfate 1.17 mmol/L, calcium chloride 2.5 mmol/L, potassium phosphate 1.18 mmol/L, HEPES 10 mmol/L, and glucose 5.5 mmol/L) and maintained at a pH of 7.4 at 37°C. With the aid of a dissecting microscope, first‐order, mesenteric arterioles were isolated and removed from the surrounding fat as previously described (Novak et al. 2002). The mesenteric arterioles (unpressurized inner diameter, 100–200 *μ*m) were then transferred to a pressure arteriograph system (Living Systems, Burlington, VT).

### Evaluation of endothelium‐dependent and endothelium‐independent vasodilation

Mesenteric arteries were pretreated with EPO 50 mIU/mL, ESP 25 ng/mL, or vehicle for 20 min and preconstricted with PE to 50% initial diameter before vasodilatory responses were assessed through the cumulative addition of endothelium‐dependent vasodilators ACh (10^−9^–10^−4^ mol/L), BK (10^−9^–10^−4^ mol/L), A23187 (10^−9^–10^−4 ^mol/L), and endothelium‐independent vasodilator SNP (10^−9^–10^−4 ^mol/L) to the arteriography chamber. Our unpublished data demonstrate that Ach, BK, and A23187 elicit endothelium‐dependent vasodilatory effects in first‐order mouse mesenteric vessels through an eNOS‐dependent pathway. Vasodilation was calculated according to the following formula:

$$\text{Relaxation}\left(\%\right)=\left(D_{s}-D_{b}\right)/\left(D_{m}-D_{b}\right)\times 100$$

where *D*_m_ is the maximal inner diameter recorded at 60 mmHg in calcium‐free HEPES‐buffered PSS containing EGTA and papaverine to inhibit vascular smooth muscle function, *Ds* is the steady‐state inner diameter recorded after each addition of the drug, and *D*_b_ is the initial baseline inner diameter recorded immediately before the first addition of the vasodilatory agent.

### Evaluation of vasoconstrictor responses

To determine whether acute pretreatment with EPO, ESP, or vehicle altered vasoconstrictor responsiveness in mesenteric arterioles, concentration–response curves to PE (10^−9^–10^−4^ mol/L) and depolarizing concentration of K^+^ (0 mmol/L–100 mmol/L) were evaluated as previously described (Muller‐Delp et al. 2002). Briefly, intraluminal diameters were measured in micrometers at 60 mmHg and expressed as a percentage of vasoconstriction as follows:

$$\text{Vasoconstriction}\left(\%\right)=\left(D_{b}-D_{s}\right)/D_{b}\times 100$$

where *D*_b_ was the initial baseline intraluminal diameter measured before experimental intervention and *D*_s_ was the steady‐state intraluminal diameter measured after agonist addition.

### Statistical and data analysis

Two‐way repeated‐measures ANOVA was used to detect differences between (vehicle vs. treatment) and within (drug concentrations) factors for the mesenteric resistance artery studies (GraphPad Software, La Jolla, CA). All data are presented as means ± SE. In all statistical analyses, *n* indicates the number of animals in each group. Student's *t*‐tests were performed to identify differences between the maximal responses (*E*_max_) to ACh, SNP, BK, A23187, KCl, and PE to identify differences in the amount of dilation with respect to intervention, and to determine differences in sensitivity (EC_50_) to ACh, SNP, BK, A23187, KCl, and PE. Significance was defined as *P* ≤ 0.05.

## Results

### EPO and ESP impair ACh‐mediated endothelium‐dependent vasodilation but not endothelium‐independent vasodilation by SNP

**Figure 1:**
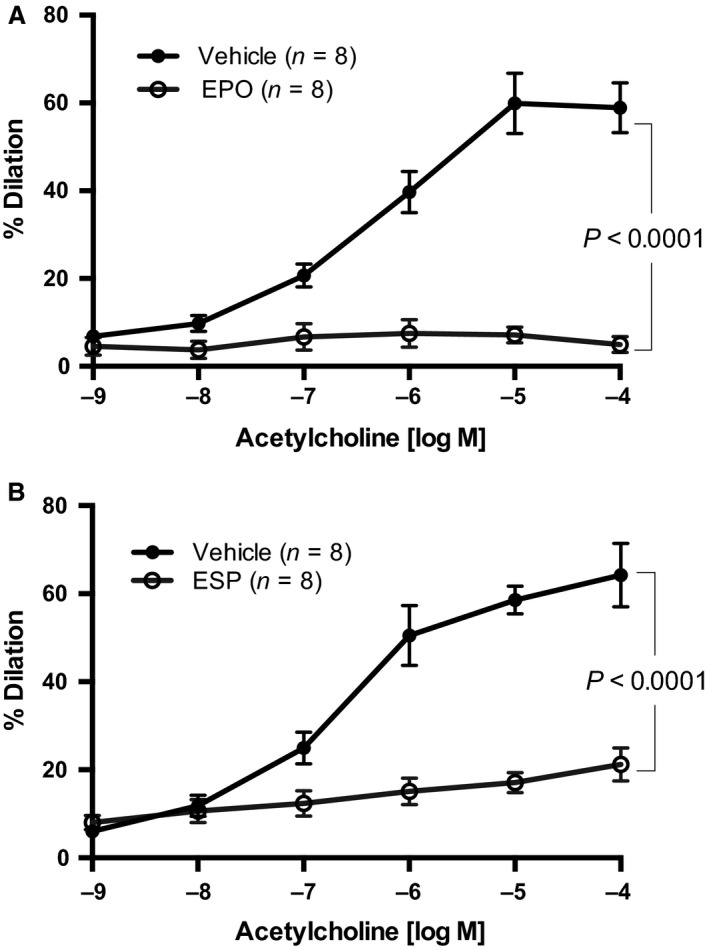
Effect of EPO or the ESP on ACh‐induced vasodilation of first‐order mesenteric arterioles. First‐order mesenteric arteries were acutely pretreated with (A) EPO (50 mIU/mL) or (B) ESP (25 ng/mL) for 20 min before assessment of ACh‐induced endothelium‐dependent vasodilation. All values are means ± SE. *n* equals the number of animals studied.

**Figure 2:**
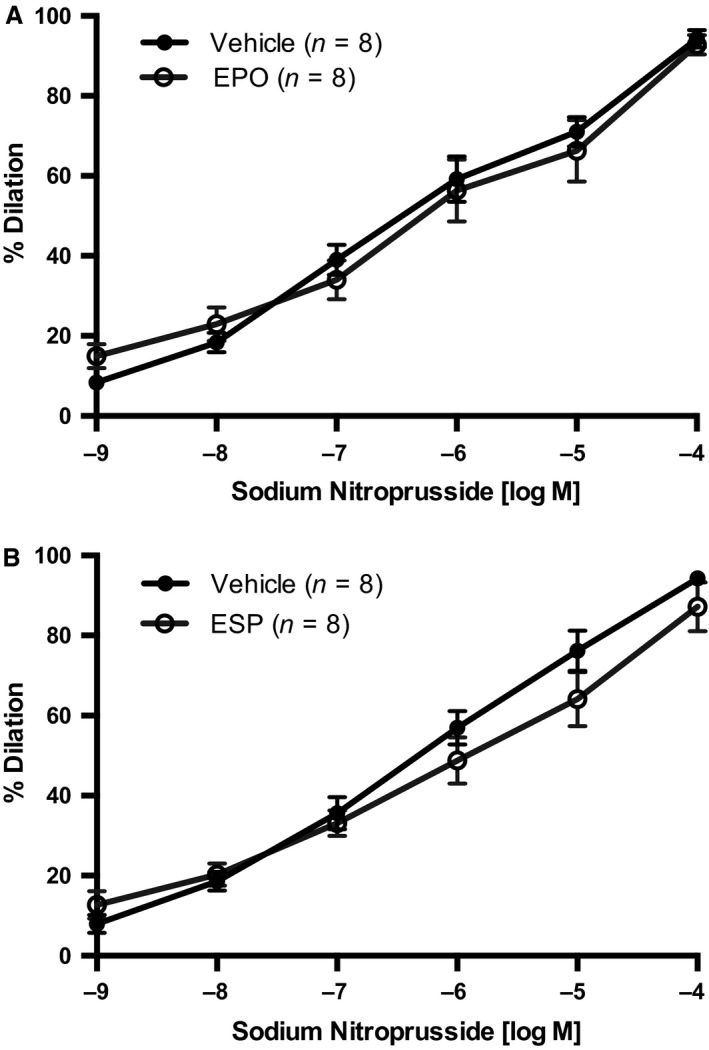
Effect of EPO and the ESP sodium nitroprusside (SNP)‐induced vasodilation. First‐order mesenteric arteries were acutely pretreated with (A) EPO (50 mIU/mL) or (B) ESP (25 ng/mL) for 20 min before assessment of SNP‐induced endothelium‐independent vasodilation. All values are means ± SE. *n* equals the number of animals studied. Responses were not different between groups.

ACh elicited a maximum vasodilation of ~60% in first‐order mesenteric arterioles (Fig. 1). However, in vessels pretreated with 50 mIU/mL EPO, the ACh‐induced vasodilation was virtually abolished (Fig. 1A). Pretreatment with 25 ng/mL ESP also impaired ACh‐induced vasodilation (Fig. 1B), but to a lesser extent than EPO (by ~60% at 10^−4 ^mol/L, respectively). Importantly, acute pretreatment with EPO or ESP did not alter endothelial‐independent vasodilation, maximal response (*E*_max_) or sensitivity (EC_50_) to SNP (Fig. 2), indicating that the two peptides did not interfere with the intrinsic ability of the vascular smooth muscle to vasodilate in response to NO. Whether the vessels were obtained from male or female mice, the response to ESP treatment was similar (results not shown).

### EPO or ESP‐induced impairment of mesenteric resistance artery endothelium‐dependent relaxation to ACh is not caused by an increase in oxidative stress

**Figure 3:**
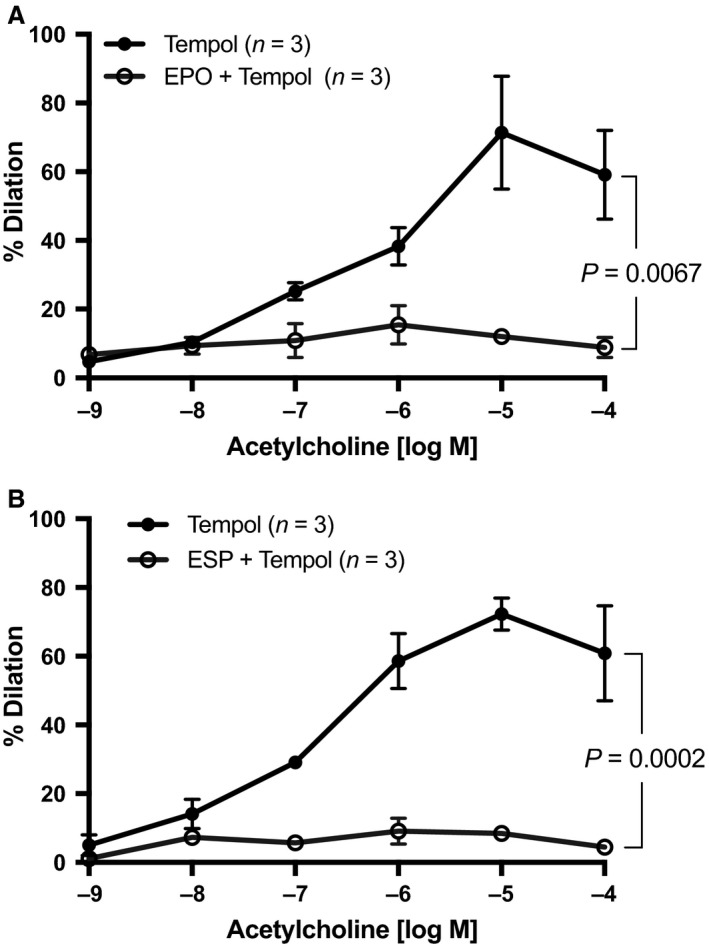
Tempol did not restore ablated ACh‐induced endothelium‐dependent vasodilation. First‐order mesenteric arteries were acutely pretreated with (A) EPO (50 mIU/mL) or (B) ESP (25 ng/mL) for 20 min in addition to Tempol (5 *μ*mol/L) before assessment of ACh‐induced endothelium‐dependent vasodilation. All values are means ± SE. *n* equals the number of animals studied.

To test whether the compromise of ACh‐induced endothelium‐dependent vasodilation by EPO and ESP was due to increased reactive oxygen species as previously reported (Chen et al. 1997), we employed the O_2_^−^ scavenger Tempol (5 *μ*mol/L). Pretreatment with Tempol for 20 min prior to EPO or ESP administration did not affect the inhibition of ACh‐induced vasodilation by EPO (Fig. 3A) or ESP (Fig. 3B).

### A23187 and bradykinin‐induced endothelium‐dependent vasodilation is unaffected by acute pretreatment with EPO or ESP

**Figure 4:**
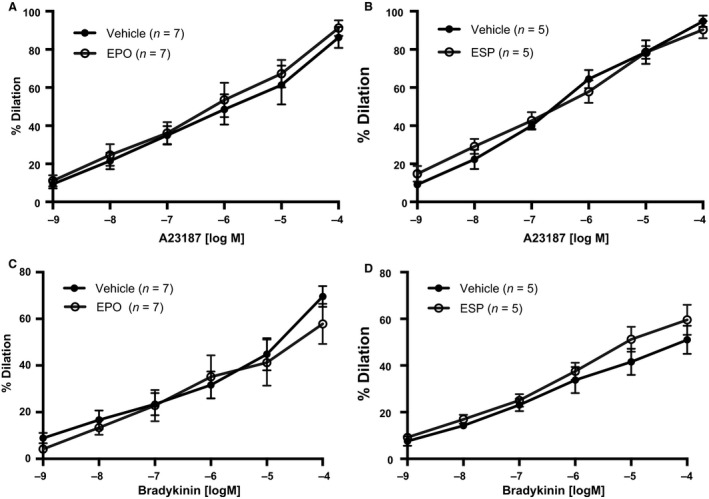
EPO or ESP had no effect on endothelium‐dependent relaxation to A23187 or BK. First‐order mesenteric arteries subjected to acute pretreatment of EPO (50 mIU/mL) (A, C) or an ESP (25 ng/mL) (B, D) before endothelium‐dependent vasodilators A23187 and BK were assessed. All values are means ± SE. *n* equals the number of animals studied.

To determine whether the effect of EPO and ESP was unique to ACh, we investigated bradykinin and A23187 – receptor‐mediated and nonreceptor‐mediated endothelium‐dependent vasodilators, respectively. On one hand, ACh and bradykinin receptors both utilize G‐protein coupled inositol‐1,4,5‐trisphosphate (IP_3_) signaling cascade in endothelial cells to release calcium from endoplasmic reticulum that, in turn, activates K^+^ channels leading to hyperpolarization and calcium influx (Brenner et al. 1989). On the other hand, A23187 directly increases cell permeability to calcium, thereby stimulating eNOS. Pretreatment of first‐order mesenteric arterioles with EPO or an ESP had no effect on A23187‐ or bradykinin‐induced endothelium‐dependent vasodilation suggesting that EPO inhibits ACh‐induced vasodilation at the level of muscarinic receptor or muscarinic receptor‐G protein interaction (Fig. 4A and B). Additionally, (EC_50_) and maximal response (*E*_max_) were unaffected in vessels pretreated with EPO or ESP when compared with controls in both A23187‐ or BK‐induced vasodilation.

### *β*IP prevents inhibition of ACh‐induced endothelium‐dependent vasodilation by EPO or ESP

**Figure 5:**
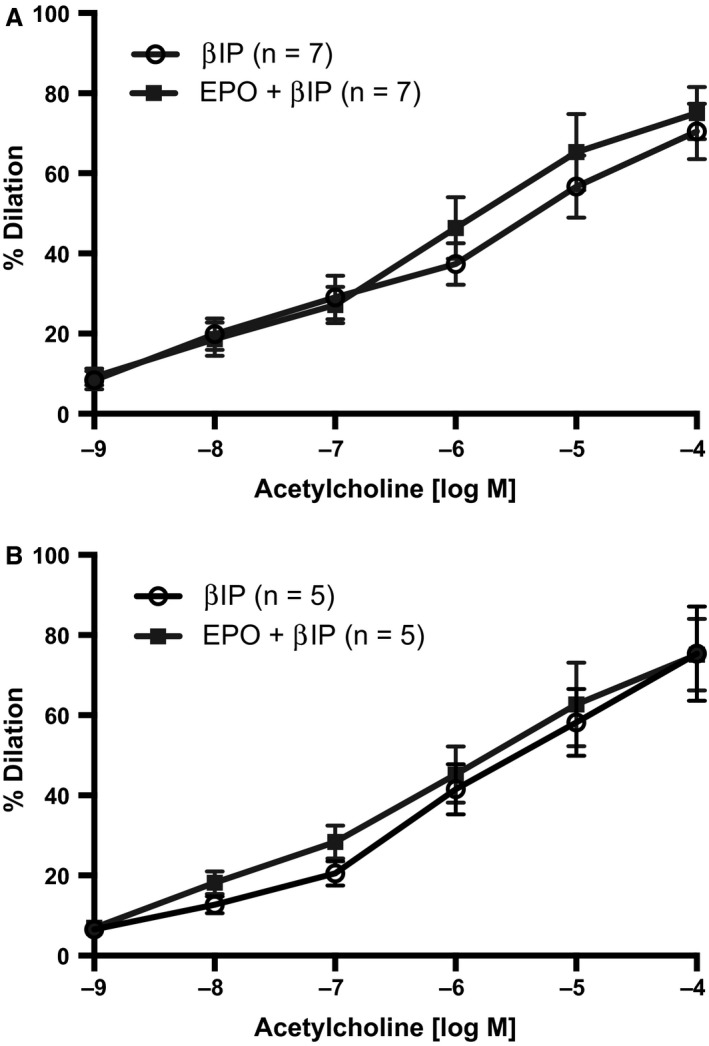
Novel *β* IP restores ablated ACh‐induced endothelium‐dependent vasodilation. First‐order mesenteric arteries were treated with the *β* IP (10 *μ*mol/L) for 30 min prior to being acutely pretreated with (A) EPO (50 mIU/mL) or (B) ESP (25 ng/mL) for 20 min. ACh‐induced endothelium‐dependent vasodilation was then assessed. Sensitivity of EPO (EC_50_ = 1.758 × 10^−6^) or ESP (EC_50_ = 2.017 × 10^−6^) was not reduced when compared with vehicle or when compared with one another. All values are means ± SE. *n* equals the number of animals studied. Responses were not different between groups.

Pretreatment of mesenteric arteries with *β*IP blocked the inhibition of ACh‐induced vasodilation in vessels by EPO (Fig. 5A) or ESP (Fig. 5B). Additionally, there was also no significant differences between the sensitivity of EPO (EC_50_ = 1.758 × 10^−6^) or ESP (EC_50_ = 2.017 × 10^−6^) to ACh‐induced vasodilation. Moreover, the maximal responses (*E*_max_) were also not significantly different.

### EPO or ESP does not alter receptor‐dependent or receptor‐independent vasoconstriction

**Figure 6:**
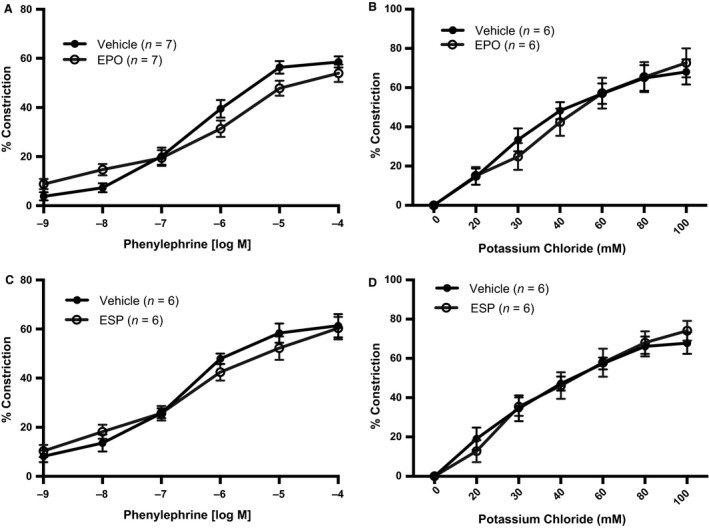
Comparison of PE (A, C) and KCl (B, D) constrictor responses with acute (20‐min) pretreatment of EPO (50 mIU/mL) (A, B) and the ESP (25 ng/mL) (C, D) All values are means ± SE. *n* equals the number of animals studied. Responses were not different between groups.

Because it has been reported that EPO increases production of potent vasoconstrictors, such as endothelin‐1 (Vogel et al. 1997; Barhoumi et al. 2014), we explored whether acute pretreatment with EPO or ESP would potentiate vasoconstriction in first‐order mesenteric arteries. Neither EPO nor ESP, however, affected vasoconstriction by PE (Fig. 6A and C) or KCl (Fig. 6B and D). Additionally, EPO or ESP did not alter the sensitivity (EC_50_) or maximal response (*E*_max_) of the vasoconstrictors PE or KCl.

## Discussion

There are several new findings that emerged from this research: (1) acute activation of the *β*CR with EPO or ESP in mouse mesenteric arteries inhibited ACh, but not BK or A23187 endothelium‐dependent vasodilation, and this inhibition of ACh was blocked by a novel *β*CR inhibitor, *β*IP; (2) impaired ACh‐induced vasodilation by EPO and ESP did not appear to be due to generation of reactive oxygen species; and (3) activation of the *β*CR by either EPO or ESP did not affect mesenteric vessel vasoconstriction to PE and KCl. Our results suggest that activation of the *β*CR on the vascular endothelium with EPO or ESP specifically impairs endothelial vasodilatory responses to ACh, which may contribute to the pathogenesis of EPO‐induced hypertension.

Animal models have been utilized to try to investigate the clinical observation of EPO‐induced hypertension (Vaziri et al. 1995, 1996; Schiffl and Lang 1997; Vaziri 1999). Animals given high doses of EPO exhibit a marked increase in hemoglobin concentration (Vaziri 1999; Singh et al. 2006; Fishbane and Besarab 2007), as well as increased systemic vascular resistance (Becker et al. 2016) and blood pressure (Buemi et al. 1995; Kanbay et al. 2007; Becker et al. 2016) similar to patients. In addition to increased systemic vascular resistance and blood pressure, small resistance arteries demonstrate diminished endothelium‐dependent vasodilation to ACh (Buemi et al. 1995; Noguchi et al. 2001; Annuk et al. 2006; Briet et al. 2013). However, there are no reports on whether EPO treatment differentially affects receptor‐ and nonreceptor‐mediated endothelium‐dependent vasodilation, and if so, which of the EPO receptor dimeric complexes might be involved. Our data demonstrate that after short‐term incubation with EPO or an ESP, ACh‐induced vasodilation is selectively impaired via the EPOR/*β*CR heterodimeric receptor (Fig. 1).

Briet et al. (2013) demonstrated that oxidative stress plays a role in EPO‐induced hypertension in humans, and in gluteal subcutaneous resistance arteries isolated from these patients, Tempol restored endothelial function. However, in our model, Tempol did not ameliorate the inhibition of ACh‐mediated vasodilation by EPO or ESP in isolated mouse mesenteric arteries (Fig. 3), suggesting that the impairment was not due to increased reactive oxygen species. Although there are many potential explanations for these discrepant results, one possibility is the marked difference in the duration of arterial exposure to EPO (months in vivo vs. minutes in vitro).

We further demonstrated an essential role for the *β*CR in the impairment of ACh‐mediated vasodilation by EPO, insofar as ESP, which specifically agonizes the *β*CR/EPOR_2_ heterodimer and does not stimulate erythropoiesis, duplicated the inhibitory action of EPO, albeit perhaps to a lesser degree. To our knowledge, there are limited reports of the involvement of this receptor in endothelial function (Su et al. 2011). We confirmed the critical role of *β*CR by using a novel inhibitor of *β*CR (*β*IP), which acts as a competitive inhibitor of ligand binding to *β*CR. *β*IP abrogated the ability of EPO and ESP to inhibit the vasodilatory effect of ACh. Interestingly, EPO and ESP had similar sensitivities (EC_50_) to ACh after treatment with *β*IP, suggesting that activation of the *β*CR is responsible for decreasing the vasoactive response to ACh.

The mechanisms by which EPO compromises ACh‐mediated endothelium‐dependent vasodilation is unknown. Using immunoprecipitation and immunofluorescence, our group reported co‐localization of *β*CR and vascular endothelial growth factor receptor 2 (VEGFR‐2) in human bone marrow‐derived angiogenic cells (BMDACs) (Sautina et al. 2010), and other investigators also reported that muscarinic acetylcholine receptor (mAChR) has a physical interaction with VEGFR‐2 in human neuroblastoma cells (Edelstein et al. 2011). Furthermore, both VEGFR‐2 and mAChR have been shown to localize in caveolae (Rybin et al. 2000; Cho et al. 2004; Sonveaux et al. 2004; Lampugnani et al. 2006). Taken together, these findings raise the possibility that *β*CR and the muscarinic acetylcholine receptor 2 (mAChR2) are co‐localized in the endothelial caveolae. It has been suggested that once activated, the *β*CR is internalized to elicit its downstream responses (Debeljak et al. 2014). However, there is no experimental evidence to support this mechanism. Further studies are needed to determine if *β*CR is associated with the caveolae in endothelial cells, and if so, whether activation leads to internalization. How this might lead to impairment of ACh‐mediated endothelium‐dependent vasodilation also requires further investigation.

Carlini et al. (1993b) have shown that animals treated with EPO have increased expression of ET‐1 (Su et al. 2011). Also, EPO administration to patients with anemia lead to increased production of cyclooxygenase‐dependent vasoconstrictors in forearm‐resistance arteries (Carlini et al. 1993a). Bode‐Böger et al. (1996) described that acute EPO treatment significantly enhanced noradrenaline‐induced vasoconstriction in rabbit aorta and carotid arteries. However, our data suggest that after acute treatment in vitro, EPO does not potentiate vasoconstrictor responses in mouse mesenteric arteries or reduce baseline internal diameter, thus mitigating against a role for cyclooxygenase‐generated vasoconstrictors. One major difference between the studies described above and this current study is the concentration of EPO used for experimentation, which was 100–1000 times the concentration that was used in this study (Bode‐Böger et al. 1996).

In summary, we demonstrated that activation of the *β*CR leads to impairment of ACh‐induced vasodilation, which is prevented by a novel *β*IP. To our knowledge, this is the first study showing that activation of the *β*CR affects vasoreactivity. In the current literature, little evidence exists for the role of the *β*CR on the vasculature (Bohr et al. 2013). *β*CR knockout mice appears to have normal development and, as would be expected, normal erythropoiesis (Sautina et al. 2010; Hand and Brines 2011; Cerami 2012; Debeljak et al. 2014; Collino et al. 2015). However, investigation of blood pressure and arterial function has not been reported. It would also be interesting to test whether *β*IP attenuates EPO‐induced hypertension in experimental animal models.

## Conflict of Interest

None declared.
